# Turbulence in protein folding: Vorticity, scaling and diffusion of probability flows

**DOI:** 10.1371/journal.pone.0188659

**Published:** 2017-12-05

**Authors:** Vladimir A. Andryushchenko, Sergei F. Chekmarev

**Affiliations:** 1 Institute of Thermophysics, SB RAS, Novosibirsk, Russia; 2 Department of Physics, Novosibirsk State University, Novosibirsk, Russia; University of Leeds, UNITED KINGDOM

## Abstract

Recently, when studying folding of a SH3 domain, we discovered that the flows of transitions between protein states can be surprisingly similar to turbulent fluid flows. This similarity was not restricted by a vortex pattern of the flow fields but extended to a spatial correlation of flow fluctuations, resulting, in particular, in the structure functions such as in the Kolmogorov theory of homogeneous and isotropic turbulence. Here, we undertake a detailed analysis of spatial distribution of folding flows and their similarity to turbulent fluid flows. Using molecular dynamics simulations, we study folding of another benchmark system—Trp-cage miniprotein, which has different content of secondary structure elements and mechanism of folding. Calculating the probability fluxes of transitions in a three-dimensional space of collective variables, we have found that similar to the SH3 domain, the structure functions of the second and third orders correspond to the Kolmogorov functions. The spatial distributions of the probability fluxes are self-similar with a fractal dimension, and the fractal index decreases toward the native state, indicating that the flow becomes more turbulent as the native state is approached. We also show that the process of folding can be viewed as Brownian diffusion in the space of probability fluxes. The diffusion coefficient plays a role of the key parameter that defines the structures functions, similar to the rate of dissipation of kinetic energy in hydrodynamic turbulence. The obtained results, first, show that the very complex dynamics of protein folding allows a simple characterization in terms of scaling and diffusion of probability fluxes, and, secondly, they suggest that the turbulence phenomena similar to hydrodynamic turbulence are not specific of folding of a particular protein but are common to protein folding.

## Introduction

Protein folding and hydrodynamic turbulence are two challenging problems that attract attention of researchers for years. Turbulent motion of a fluid is a stochastic motion, which arises due to the instability of the fluid flow at large Reynolds numbers, i.e., when the inertia of the fluid motion dominates over viscosity [1–4]. Typically, the turbulent motion appears as a cascade of eddies of various sizes. One example is when large eddies generated by external forces, e.g., by the walls of the pipe through which the fluid flows, disintegrate into smaller eddies until the latter dissipate due to viscosity (the Richardson cascade [5]). In contrast to the fluid, which is a collection a large number of atoms (∼ 10^24^) and thus can be described in the macroscopic terms such as the average velocity, density, etc., a protein is a system of finite size, which can be as small as of ∼ 10^3^ atoms, and thus requires the description on atomic level. Synthesized on the ribosome as a chain of amino acid residues, a protein folds into a compact functional (native) state. The process of folding is typically very complex, with a variety of folding pathways and metastable states [6–11]. One essential feature of protein folding that makes it similar to hydrodynamic turbulence [1–5] is that the process of folding is inherently a cascade process—in the present case, in the form of sequential rearrangement of the protein structure from an unfolded state to the native state. The cascade nature of the process is also characteristic of the other known types of turbulence—the wave [12], market [13] and superfluid [14] turbulence (see, also, a discussion of the cascades in the latter case [15]).

A detailed analysis of similarity between protein folding and hydrodynamic turbulence becomes possible if, instead of evolution of protein structure in the multidimensional (all-atom) conformational space, we consider probability fluxes of transitions between characteristic states of the protein in a reduced space of collective variables. Such is a recently proposed hydrodynamic description of protein folding [16]. The purpose of that approach was to gain a closer insight into folding dynamics, because typically employed free energy surfaces (FESs) display only the probability for the protein to be in a current state but do not show the direction in which the protein proceeds (folds, unfolds, or dwells in the current state). The process of “first-passage folding”, i.e., when the folding trajectories are initiated in a unfolded state of the protein and terminated upon reaching the native state, is of particular interest because it corresponds to physiological conditions when the native state is stable and unfolding events are improbable [17]. Having the probability fluxes, the process of first-passage folding can be viewed as a stationary flow of a “folding fluid” from an unfolded state of the protein to its native state, with the density of the fluid being proportional to the probability for the system to be in the current state. The analysis of the first-passage folding of several model proteins (an *α*-helical hairpin [16], a SH3 domain [18, 19], and beta3s [17, 20, 21] and 2evq [22] miniproteins) has shown that the folding flows do not generally follow the FESs and typically contain vortices that remind eddies in turbulent flows. To see how the protein folding flows are close to turbulent fluid flows, the folding flows of SH3 domain were characterized in terms accepted in hydrodynamic turbulence [19]. Specifically, there were calculated so called structure functions, which represent velocity space correlation functions [2], or, more exactly, flux space correlation functions, because the folding fluid is highly “compressible” [19]. According to the Kolmogorov theory of isotropic and homogeneous turbulence (K41) [23, 24], the fluctuations of the flow velocities scale with the space increment *l* as *l*^1/3^, so that the structure functions of the second and third order vary as *l*^2/3^ and *l*, respectively. Very surprisingly, it was found that the corresponding structure functions for folding flows of SH3 domain reveal exactly the same dependence on the increment in the inter-residue contact space [19].

These results for SH3 domain lead to a natural question of how such turbulence phenomena are common to protein folding. To see that, we consider another benchmark system—the Trp-cage miniprotein [25–32], whose secondary structure content and mechanism of folding are essentially different from those for SH3 domain. In particular, the kinetics of Trp-cage folding are single-exponential, while folding kinetics of SH3 domain were double-exponential, and the turbulent flow was observed only for slow folding trajectories [19]. Also, we employ an essentially different approach to study the Trp-cage folding: First, the molecular dynamics (MD) simulations are performed using an all-atom model (CHARMM program [33]), while for the SH3 domain a coarse-grained representation of the protein was used, in which the amino acid residues were considered as monomers placed on positions of C_*α*_-atoms in the protein chain (C_*α*_-model) [19]. Secondly, the collective variables are determined with a principal component analysis (PCA) method [34], while in the case of SH3 domain they were represented by weakly dependent groups of native contacts, which were considered as “physically” orthogonal variables [19]. We find that despite such a difference between these proteins and their characterization, the structure functions of the second and third orders for the Trp-cage follow the Kolmogorov scaling for isotropic and homogeneous turbulence, similar to those in the case of SH3 domain. Further, we show that the time-rate of change of the variance of folding fluxes is approximately constant in the dominant interval of times, so that it can be considered as a key parameter to characterize folding flows, similar to the rate of energy dissipation in hydrodynamic turbulence. Accordingly, the process of protein folding can be viewed as Brownian diffusion in the space of probability fluxes. Finally, we show that the folding flows are self-similar with a fractal dimension, and the fractal index decreases as the native state is approached.

The paper is organized as follows. The next section briefly describes the protein model, the simulation technique, the methods we used to characterize the folding process, and a general picture of Trp-cage folding (for more details see [32]). The subsequent section presents the results and their discussion. The last section contains concluding remarks.

## Folding of Trp-cage miniprotein

### System and simulation method

**Fig 1:**
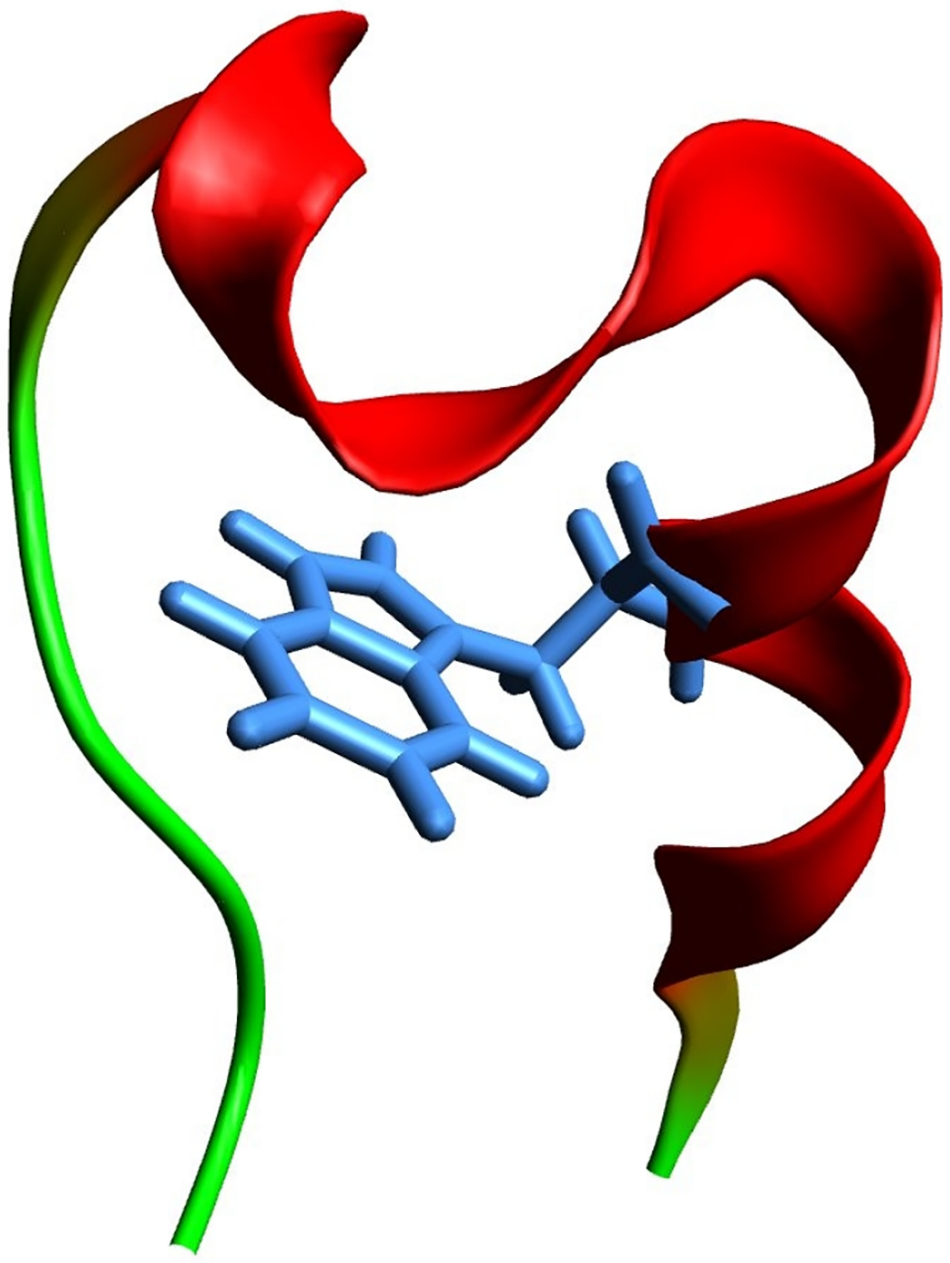
The native structure of the Trp-cage miniprotein (1L2Y.pdb) in a ribbon representation. The Trp6 residue is shown in blue sticks.

Trp-cage is a 20-residue miniprotein (Asn1-Leu2-Tyr3-Ile4-Gln5-Trp6-Leu7-Lys8-Asp9-Gly10-Gly11-Pro12-Ser13-Ser14-Gly15-Arg16-Pro17-Pro18-Pro19-Ser20; 1L2Y.pdb) [25]. It consists of a N-terminal *α*-helix (residues 2-8), a 3_10_-helix (residues 11-14), and a C-terminal polyproline II (PPII) helix (residues 17-19), which form a hydrophobic core with the Trp6 buried in the center (Fig 1). The interactions between Tyr3, Trp6, Gly11, Pro12, Pro18, and Pro19 lead to formation of the Trp-cage fold. To perform MD simulations, the CHARMM program [33] was employed. All heavy atoms and the hydrogen atoms bound to nitrogen or oxygen atoms were considered explicitly; PARAM19 force field [35] and a default cutoff of 7.5Å for the nonbonding interactions were used. To take into account the main effects of the aqueous solvent, a meanfield approximation based on the solvent-accessible surface (SAS) [36] was employed. The simulations were performed with the time step of 2 fs using the Berendsen thermostat (coupling constant of 5 ps) [37]. Although such an approach overestimates folding rates, mostly because of the absence of the friction of protein atoms against the solvent, the relative rates of formation of secondary structural elements are comparable to the values observed in experiment; i.e., *α*-helices fold in about 1 ns and *β*-hairpins in about 10 ns [38] compared to experimental values of ∼ 0.1*μ*s and ∼ 1*μ*s [39], respectively.

The folding trajectories were initiated in unfolded states of the protein and terminated upon reaching the native state. The unfolded states were prepared using the standard CHARMM protocol [33]; i.e., an extended conformation of the protein was first minimized (200 steps of the steepest descent followed by 300 steps of the conjugate gradient algorithm) and then heated to *T* = 300K and equilibrated for 5 × 10^3^ time steps. A native contact was assumed to be formed if the distance between the C_*α*_-atoms in the residues which are not neighbors in the sequence is less than 6.5Å in all NMR structures [25], which resulted in 35 native contacts. The simulations were conducted for *T* = 300K; at this temperature, the mean first-passage time (MFPT) was minimal and equal to ≈ 36ns, which is in good agreement with the experimental time (4.1*μ*s [26]), if to take into account that the simulations with implicit solvent overestimate the rate of formation of secondary structure elements by ≈ 10^2^ times. There were simulated 100 folding trajectories. Protein conformations (“frames”) were stored each 20 ps, which resulted in 229420 conformations in total.

### Conformation space and collective variables

To characterize protein conformations, the distances between C_*α*_-atoms in the residues that formed native contacts were used. This space of the distances was then transformed to a space of orthogonal collective variables using the PCA method [34]. It was found that the first three eigenvalues were well separated from the others and captured ≈ 29%, ≈ 21% and ≈ 19% of the data variation, which resulted in ≈ 69% of information in total. The eigenvectors corresponding to these modes were chosen to form a three-dimensional (3D) space of collective variables **g** = (*g*_1_, *g*_2_, *g*_3_). To determine a two-dimensional (2D) space of variables, **G** = (*G*_1_, *G*_2_), the variable *G*_1_ was chosen as *g*_1_, and the variable *G*_2_ was determined as a sum of the second and third eigenvectors weighted according to their eigenvalues, similar to Ref. [22]. Since the collective variables are linear combinations of the original variables (distances), they are measured in the same units as the latter, specifically, in angstroms.

### Probability fluxes

The probability fluxes, determining the local rates of transitions between protein states in the **g** space, were calculated according to the hydrodynamic description of protein folding [16]. In the case of the 3D space of collective variables, the *g*_1_-component of the flux **j**(**g**) was calculated as

$$j_{g_{1}}\left(g\right)=[\sum_{g^{\prime },g^{\prime \prime }\left(g\subset g^{*}\right)}^{g_{1}^{\prime \prime }-g_{1}^{\prime }>0}\;n\left(g^{\prime \prime },g^{\prime }\right)\;-\;\sum_{g^{\prime },g^{\prime \prime }\left(g\subset g^{*}\right)}^{g_{1}^{\prime \prime }-g_{1}^{\prime }<0}\;n\left(g^{\prime \prime },g^{\prime }\right)]/\left(M{\bar{t}}_{f}\Delta g_{2}\Delta g_{3}\right)$$ (1)

where *M* is the total number of simulated trajectories, ${\bar{t}}_{f}$ is the MFPT, *n*(**g**′′, **g**′) is the number of transitions from state **g**′ to **g**′′, and **g** ⊂ g* is a symbolic designation of the condition that the transitions included in the sum have the straight line connecting points **g**′ to **g**′′, which crosses the plane *g*_1_ = const within the elementary cell Δ*g*_2_ × Δ*g*_3_ centered at the point **g**. The *g*_2_ and *g*_3_ components of **j**(**g**) are determined in a similar way, except that one selects the transitions crossing the planes *g*_2_ = const and *g*_3_ = const within the cells Δ*g*_1_ × Δ*g*_3_ and Δ*g*_1_ × Δ*g*_2_, respectively. In the case of the 2D space, the planes and elementary cells are replaced with the lines and elementary segments along these lines, respectively. The calculations were performed on a grid with discretization Δ*g*_1_ = Δ*g*_2_ = Δ*g*_3_ = 1Å. In what follows, distances and times will be measured in angstroms and microseconds, respectively.

### Visualization of the streamlines

To visualize the streamlines in the 3D space of variables, **g** = (*g*_1_, *g*_2_, *g*_3_), we used passive tracers. Starting from various points of the **g** space, there was solved the equation

$$\begin{array}{c} \frac{dg}{d\tau }=j\left(g\right) \end{array}$$ (2)

where **j**(**g**) is determined by Eq (1), and *τ* is a parameter (“time”). To calculate intermediate values of **j**(**g**), an algorithm of linear interpolation between the neighboring points [40] was used.

In the case of the 2D space of variables, **G** = (*G*_1_, *G*_2_), the streamlines can be calculated as the lines corresponding to constant values of the stream function [2]

$$\begin{array}{c} \Psi \left(G_{1},G_{2}\right)=\int_{G_{2}^{\prime }=0}^{G_{2}^{\prime }=G_{2}}\;J_{G_{1}}\left(G_{1},G_{2}^{\prime }\right)dG_{2}^{\prime } \end{array}$$ (3)

where **J**(**G**) is the probability flux in the 2D space. Then, two streamlines that satisfy the equations Ψ(*G*_1_, *G*_2_) = *C*_1_ and Ψ(*G*_1_, *G*_2_) = *C*_2_, where the constant *C*_1_ and *C*_2_ obey the condition *C*_2_ > *C*_1_, create a stream tube which contains the (*C*_2_ − *C*_1_)/Π fraction of the total flow $\Pi =\int \;J_{G_{1}}\left(G_{1},G_{2}^{\prime }\right)dG_{2}^{\prime }$.

### General picture of Trp-cage folding

**Fig 2:**
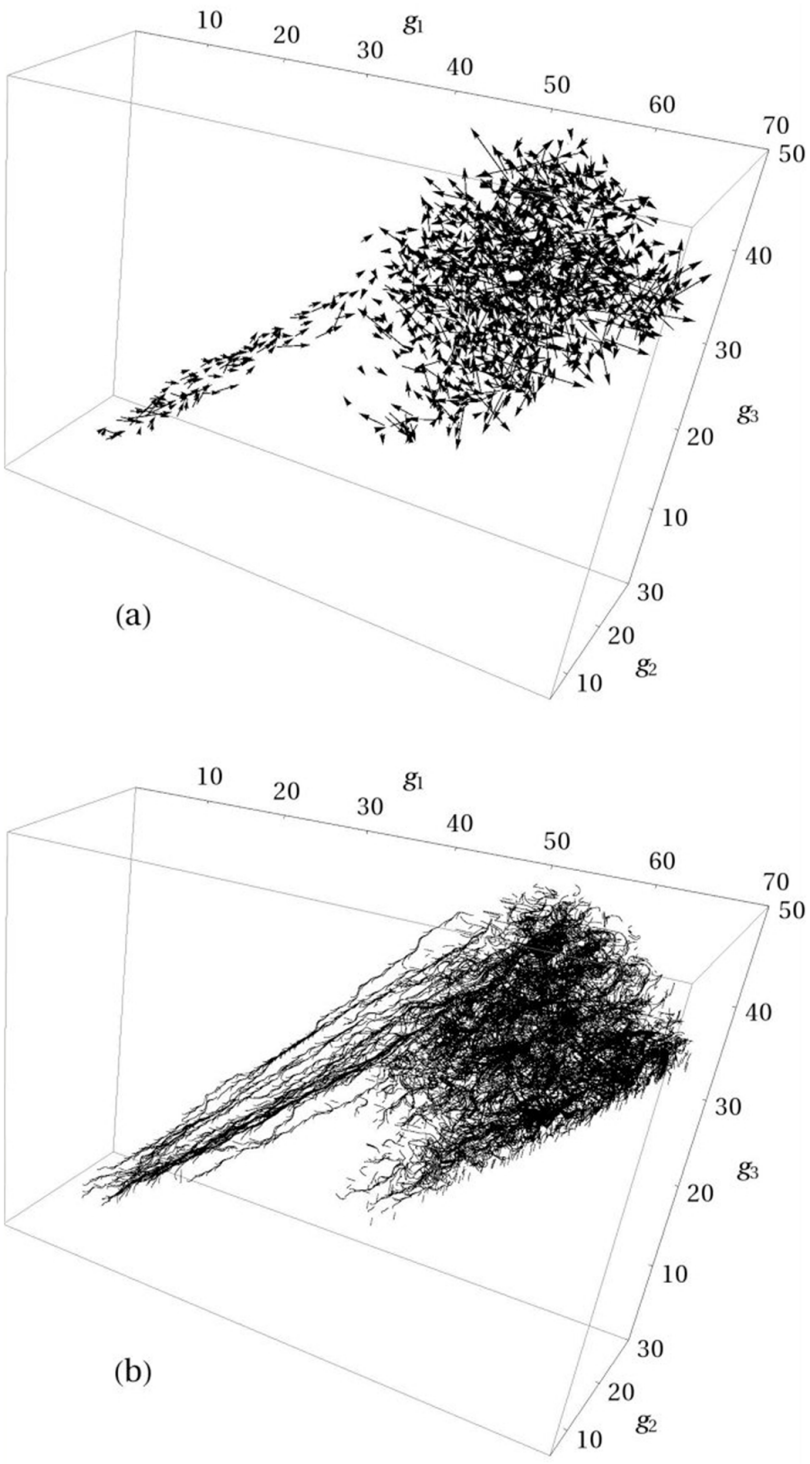
Three-dimensional flow field of the Trp-cage folding. Panel (**a**) shows the vector flow field, and panel (**b**) depicts the folding trajectories in the form of passive tracers (for illustration purpose, twenty randomly selected trajectories were chosen). Folding trajectories are initiated in the region of unfolded states (*g*_1_ ≈ 12.0, *g*_2_ ≈ 14.0, *g*_3_ ≈ 7.0) and terminated in the native state (*g*_1_ ≈ 65.5, *g*_2_ ≈ 14.8, *g*_3_ ≈ 38.1).

**Fig 3:**
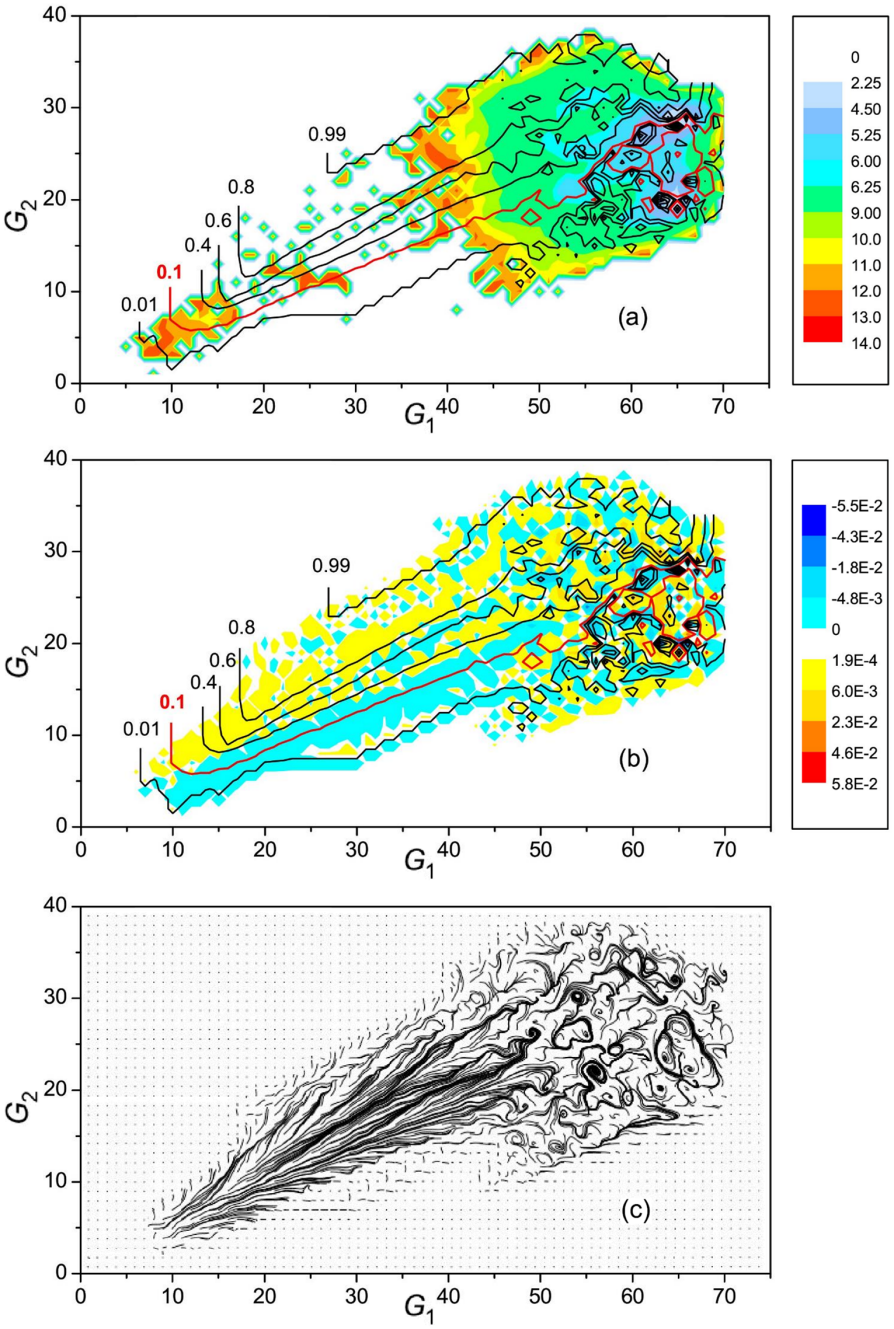
Two-dimensional flow field. Streamlines of the folding flows superimposed on (**a**) the free energy surface and (**b**) the vorticity distribution, and (**c**) folding trajectories in the form of passive tracers. The negative vorticity corresponds to a clockwise motion, and the positive vorticity to an anti-clockwise motion. Figures at the streamlines denote the fractions of the total folding flow restricted by the current streamlines. The lowest stream tube (up to 0.1 traction of the total flow) represents pathway II, and the other stream tubes correspond to pathway I. The color scale bars at panels (**a**) and (**b**) show, respectively, the levels of the free energy and vorticity.

As has been indicated in the Introduction, the process of first-passage folding, which we consider in the present paper, can be viewed as a stationary flow of a folding fluid from an unfolded state of the protein to its native state. Fig 2**a** and 2**b** show the general picture of the flow field—the vector flow field and the folding trajectories in the form of passive tracers, respectively. The common understanding of the process of folding of Trp-cage is that it can fold trough one of two (or through both) characteristic folding pathways [28–32]: in one pathway (I), the collapse of the hydrophobic core precedes the formation of the *α*-helix, and in the other pathway (II), the *α*-helix forms first. Fig 3**a** and 3**b** show the streamlines of the folding flow superimposed, respectively, on the FES and the distribution of flow vorticity. To make the vortex picture of the flow field clearer, Fig 3**c** also presents the folding trajectories in the form of passive tracers. The free energy was calculated as

$$\begin{array}{c} F\left(G\right)=-k_{B}T\ln p\left(G\right) \end{array}$$ (4)

where *p*(**G**) is the probability for the system to be found at the point **G** = (*G*_1_, *G*_2_) and *k*_B_ is the Boltzmann constant, and the vorticity was calculated as

$$\begin{array}{c} \omega \left(G\right)=\partial J_{G_{2}}/\partial G_{1}-\partial J_{G_{1}}/\partial G_{2} \end{array}$$ (5)

**Fig 4:**
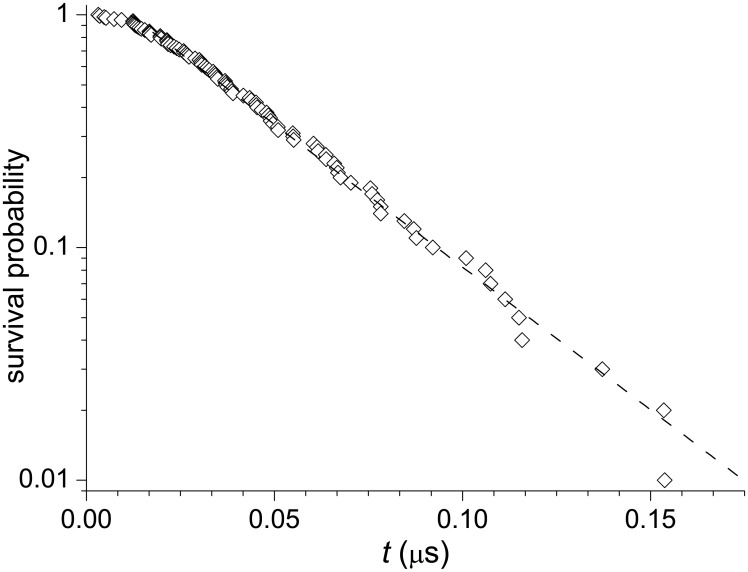
Cumulative distribution of the first-passage times. The labels correspond to the simulation results, and the dashed line is the best-fit exponential approximation with the waiting time ≈ 0.036*μ*s.

The streamlines, which divide the total folding flow from the unfolded to the native state into stream tubes, show that approximately 90% of the flow follow pathway I, in agreement with the previous MD simulation studies at *T* = 300K [28, 31]. The flow in this pathway is well directed to the native state and filled with small vortices which do not effect the general directions of the flow. In contrast, the flow in pathway II, which accounts just for 10% of the total flow, is much more complex. In particular, it contains a set of relatively large opposite-directed vortices in the region adjacent to the native state. As the previous study has shown [32], the clockwise vortices surrounding the group of anti-clockwise vortices that is centered at *G*_1_ ≈ 63 and *G*_2_ ≈ 22 form a large clockwise vortex. It is created due to a repeated partial unfolding of native-like conformations to the conformations that have a partly unformed *α*-helix and broken alignment of the *α*- and PPII-helices, which is followed by the return of the protein to a native-like state. The smaller, opposite-directed vortices within this, larger vortex, correspond to less significant changes in the protein structure; here, the rearrangements are mostly restricted to a partial forming/unforming the *α*-helix. The present complexity of the folding flows in pathway II does not lead to a considerable deviation from two-state kinetics; the distribution of the first-passage times remains essentially single-exponential (Fig 4). We note that the appearance of vortices in the flow field is not surprising [21, 41] because the condition of stationary flow (in the present case, from the source to the sink) Δ · **J** = 0 does not rule out the presence of a curl-component in **J** [42]. Such whirling flows are characterized by “irreversible circulation” or “cyclic balance”, which determine the degree of deviation from detailed balance [43–45].

## Results and discussion

In contrast to classical hydrodynamic turbulence, which considers an incompressible fluid [1–4], the folding fluid is highly “compressible” because the probability for the system to visit different points of conformation space, which plays a role of the density of the folding fluid, varies by several order of magnitude (see Eq (4) and Fig 3**a**). Therefore, to characterize the folding flow field, the probability fluxes are more suitable than the velocities [19]. This is related not only to turbulence phenomena but also to a general description of the folding flows. In particular, according to the Helmholtz decomposition theorem, a natural separation of the folding flow field into a curl-free and divergence-free vector fields is allowed, which results in a two-component potential of the driving force of protein folding [22].

**Fig 5:**
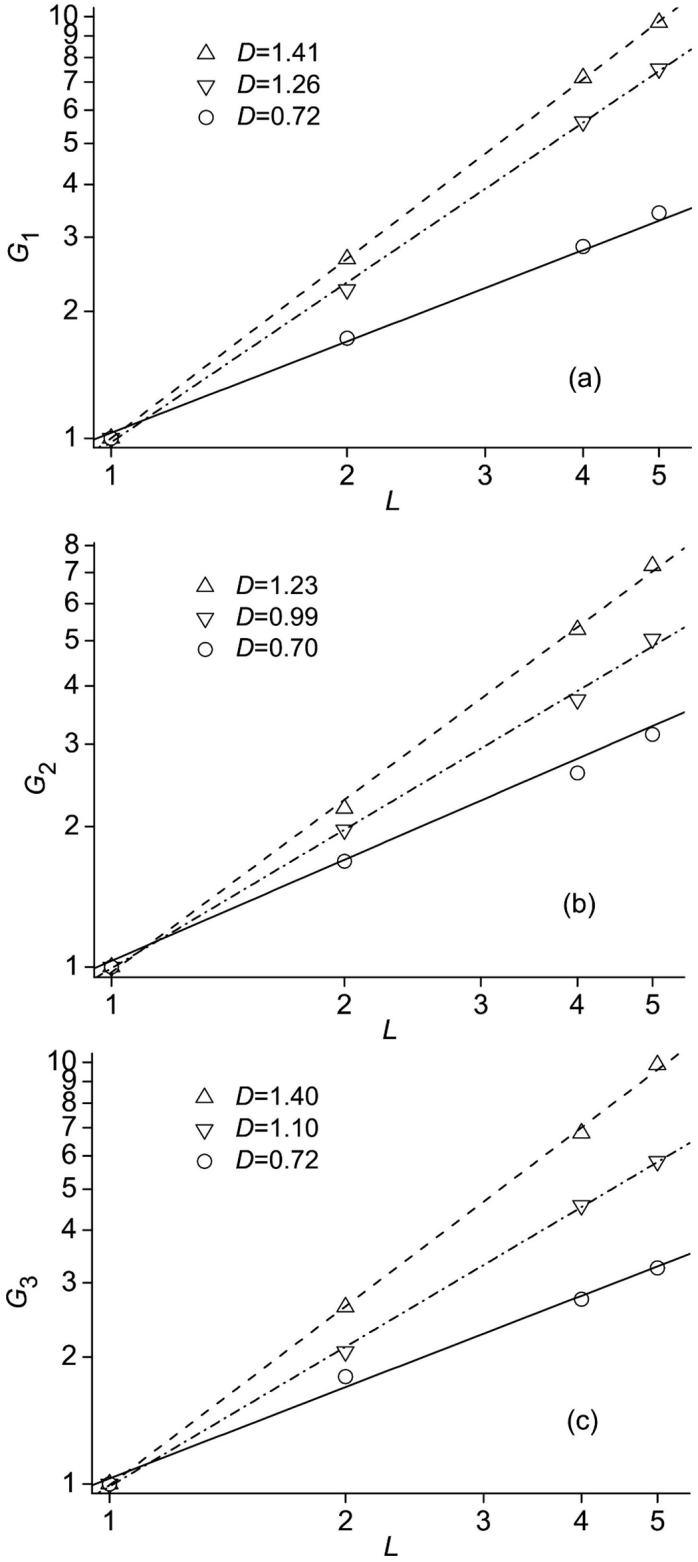
The functions *G*_*k*_(*L*) representing the *k*-components of the flow depending on the scale of coarse-graining *L*. Panels **(a)**, **(b)** and **(c)** are for *k* = 1, 2 and 3, respectively. Triangles-up correspond to the region of conformation space 10 < *g*_1_ ≤ 30, triangles-down to 30 < *g*_1_ ≤ 50, and circles to 50 < *g*_1_ ≤ 70. The lines show the best-fits of the data to the equation *G_k_*(*L*) ∼ *L*^*D_k_*^.

We start with the study of the space distribution of folding fluxes depending on the scale of spatial coarse-graining. As has been previously shown for SH3 domain [19] and beta3s miniprotein [20], although the folding flow field is far from uniform (Fig 2), the distribution of folding flows possesses a well pronounced property of self-similarity. To see if the fluxes for Trp-cage are also self-similar, and to determine the self-similarity index, we calculated the function $G_{k}\left(L\right)=<\left|J_{g_{k},L}\right|/{\bar{j}}_{g_{k}}>$, where |*J*_*g*_*k*_,*L*_| is the absolute value of *g*_*k*_ component of the flow through the square of linear size *L*, ${\bar{j}}_{g_{k}}={\left(\sum_{1}^{M}\;j_{g_{k},i}^{2}/M\right)}^{1/2}$ is the average flux in *g*_*k*_-direction through the elementary square, *M* is the number of elementary squares the square of size *L* covers, and the angular brackets denote the averaging over the *g*_*k*_ = const cross-sections of the **g** = (*g*_1_, *g*_2_, *g*_3_) space. The linear size *L* is measured in units of the elementary square linear size equal to 1Å. The maximum value of *L* was chosen to be not larger than 5Å, because the flow field is very narrow in the *g*_2_ direction; it varies from ≈ 5Åat small values of *g*_1_ to ≈ 20Åat large *g*_1_ values (Fig 2). Fig 5**a**–5**c** presents the results. In each panel, the values of *G*_*k*_(*L*) are shown for regions of conformation space that gradually shift from the unfolded to the native state along the *g*_1_ coordinate. Specifically, the triangles-up correspond to 10 < *g*_1_ ≤ 30, triangles-down to 30 < *g*_1_ ≤ 50, and circles to 50 < *g*_1_ ≤ 70. The lines show the corresponding best-fits of the data to the equation *G_k_*(*L*) ∼ *L*^*D_k_*^. It is seen that for all directions (*k* = 1, 2, 3), the flow space distributions are self-similar, and the values of *D*_*k*_ vary between approximately 0.7 and 1.4, i.e., the distributions are fractal [46]. Also, as the native state is approached, the fractal index decreases, indicating that the flow deviates from a uniform flow, for which *D* = 2, more and more. These results are in line with the previous studies of folding of SH3 domain [19] and beta3s miniprotein [17], where *D*_*k*_ decreased from ≈ 1.5 to ≈ 0.7 toward the native state.

Let us now turn to the structure functions. Specifically, we consider the conventional longitudinal functions [1–4, 23, 24], in which the increment of the flux between two points is projected on the line connecting these points. The second-order structure function is defined as

$$\begin{array}{c} C_{ll}\left(l\right)=\left\langle \delta j_{\left|\right|}{\left(l\right)}^{2}\right\rangle \end{array}$$ (6)

and the third-order function as

$$\begin{array}{c} C_{lll}\left(l\right)=\left\langle \delta j_{\left|\right|}{\left(l\right)}^{3}\right\rangle \end{array}$$ (7)

**Fig 6:**
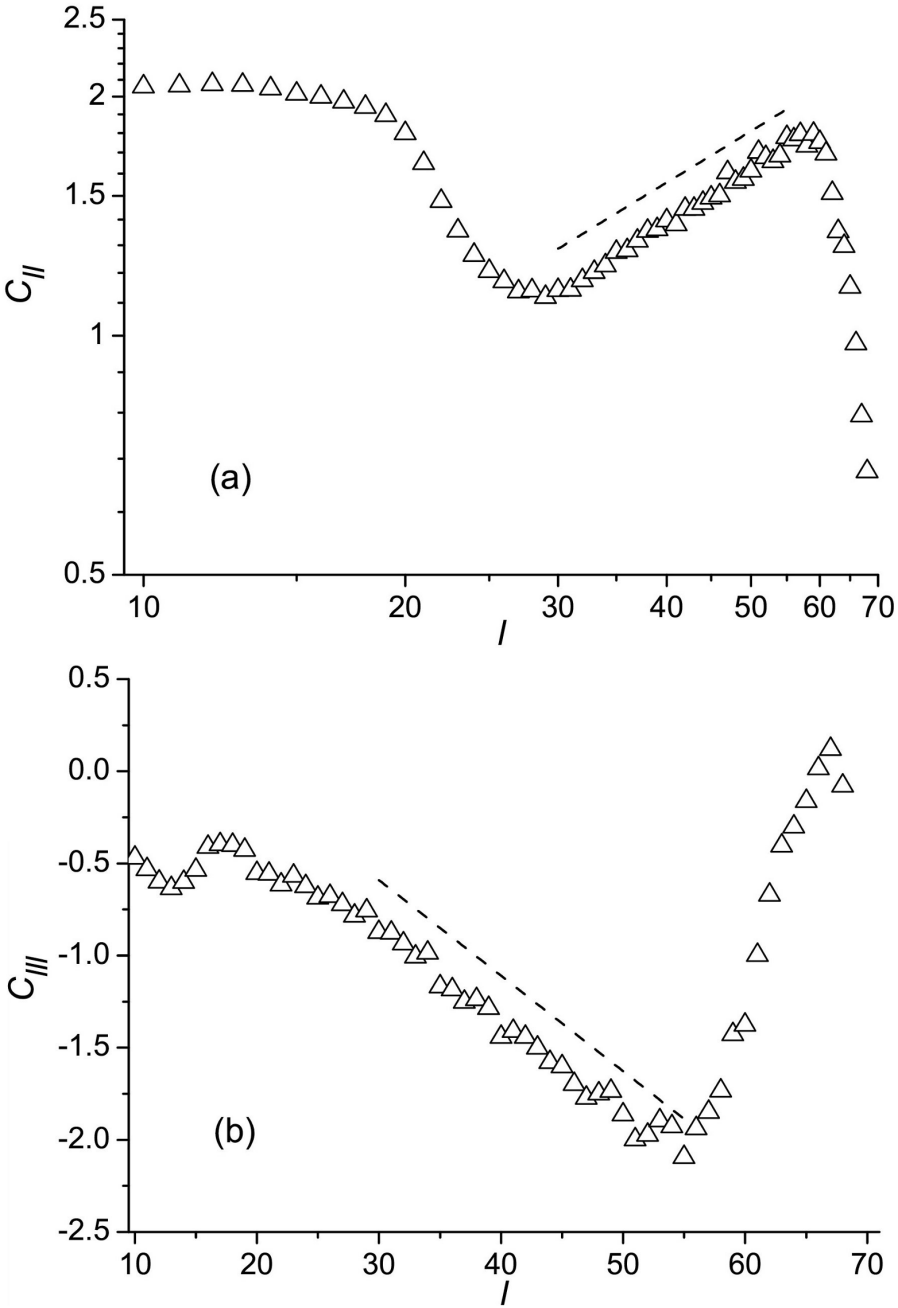
Structure functions of (a) the second and (b) third orders. The dished lines in panels (**a**) and (**b**) represent the functions *C*_*ll*_ ∼ *l*^2/3^ and *C*_*lll*_ ∼ *l*, respectively.

Here

$$\begin{array}{c} \delta j_{\left|\right|}\left(l\right)=\left[j\left(g+l\right)-j\left(g\right)\right]\;\cdot \;l/l \end{array}$$ (8)

where **l** is the increment in the **g** space, and the angular brackets denote ensemble averages. Fig 6**a** and 6**b** shows the calculated structure functions. It is seen that there is a range of space increments, approximately 30 < *l* < 55, where the functions scale with *l* as the Kolmogorov (K41) theory for isotropic and homogeneous turbulence [23, 24] predicts for the inertial interval of scales, i.e., *C*_*ll*_(*l*) ∼ *l*^2/3^ and *C*_*lll*_(*l*) ∼ *l*. The lower bound of this range is considerably larger than the characteristic distance on which the inter-residue contacts form and break (the nonbonding interaction cutoff is 7.5Å), and the upper bound is smaller than the length of the unfolded protein chain (≈ 70Å), which determines the overall size of the flow field (Fig 2). Therefore, similar to the inertial interval of scales in hydrodynamic turbulence, the only distance on which the flow increments essentially depend within the present range is the current space increment *l*.

**Fig 7:**
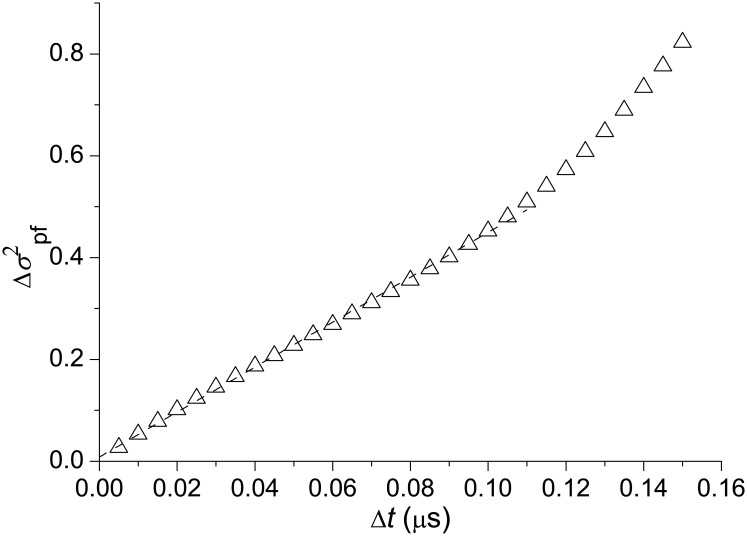
Time-dependent variance of the probability fluxes. The dashed line is the best-fit of the data for Δ*t* < 0.11*μ*s to a linear equation. To have $\Delta \sigma_{\text{pf}}^{2}$ in the same value scale with the structure functions, the volume *V*(*τ*) in Eq (10) was taken as a fraction of the maximum volume (≈ 12.2 × 10^3^ Å^3^).

According to the K41, the time-rate of change of the kinetic energy of fluctuations per unit mass *ϵ*_hd_ = *de*/*dt* is finite and constant in the inertial interval of scales, and thus it plays a role of the key parameter that determines the behavior of flow fluctuations on these scales. Then, the dimension analysis gives *δv* ∼ (*ϵ*_hd_ *l*)^1/3^ [23, 24]. The kinetic energy of flow fluctuations per unit mass is $e=(\sum \;mv_{i}^{2}/2)/M$, where *m* is the molecular mass, *v*_*i*_ is the fluid velocity in *i* point of the space volume the fluid fills, and *M* is the mass of the fluid. It can be rewritten as $e=\left(\sum \;mv_{i}^{2}/2\right)/\left(mnV\right)=\left(\sum \;v_{i}^{2}/2\right)/\left(nV\right)=n^{-3}\left(\sum \;j_{i}^{2}/2\right)/V=n^{-3}\sigma^{2}/2$, where *V* is the space volume, *n* is the (numerical) density of the fluid, *j*_*i*_ = *nv*_*i*_ is the fluid flux, and *σ*^2^ is the variance of the fluxes per unit volume. This suggests that in the case of protein folding, or more generally, in the case of compressible fluid, the time-rate of change of the variance of fluxes per unit volume $\epsilon_{\text{pf}}=d\sigma_{\text{pf}}^{2}/dt$, where $\sigma_{\text{pf}}^{2}=\langle {\left[j\left(g\right)-\langle j\left(g\right)\rangle \right]}^{2}/V\rangle$, plays a role of the key parameter, similar to *ϵ*_hd_ in hydrodynamic turbulence. Accordingly, the relation *δv* ∼ (*ϵ*_hd_*l*)^1/3^ transforms into *δj* ∼ (*ϵ*_pf_*l*)^1/3^, indicating that the flux distribution is self-similar with respect to the space increment. To perform its function, *ϵ*_pf_ should be constant. To see if this is true, we calculated the time-dependent variance of the fluxes

$$\begin{array}{c} \Delta \sigma_{\text{pf}}^{2}\left(\Delta t\right)={\left\langle \sigma_{\text{pf}}^{2}\left(t+\Delta t\right)-\sigma_{\text{pf}}^{2}\left(t\right)\right\rangle }_{t} \end{array}$$ (9)

where

$$\begin{array}{c} \sigma_{\text{pf}}^{2}\left(\tau \right)={\left\langle {\left\{j\left[g\left(\tau \right)\right]-{\left\langle j\left[g\left(\tau \right)\right]\right\rangle }_{g}\right\}}^{2}\right\rangle }_{g}/V\left(\tau \right) \end{array}$$ (10)

is the variance of the fluxes per unit volume at time *τ*, Δ*t* is the time increment, **j**[**g**(*τ*)] is the space distribution of fluxes at time *τ*, *V*(*τ*) is the volume of the **g**-space the system occupies at time *τ*, and the angular brackets denote ensemble averages over time and conformation space, which are indicated, respectively, by indices *t* and **g** at the brackets. The calculations presented in Fig 7 show that for the dominant interval of times, where statistics are not too poor (specifically at Δ*t* < 0.11*μ*s, which covers ≈ 95% of folding trajectories; see Fig 4), $\Delta \sigma_{\text{pf}}^{2}\left(\Delta t\right)$ changes with Δ*t* essentially linearly. We thus find that the time-rate of change of $\sigma_{\text{pf}}^{2}$ is approximately constant in the course of Trp-cage folding, so that the quantity $\epsilon_{\text{pf}}=d\sigma_{\text{pf}}^{2}/dt$ can be considered as a key parameter for the folding process, similar to the time-rate of change of the kinetic energy per unit mass in hydrodynamic turbulence *ϵ*_hd_. Accordingly, the structure functions *C*_*ll*_(*l*) [Eq (6)] and *C*_*lll*_(*l*) [Eq (7)] are written as

$$\begin{array}{c} C_{ll}\left(l\right)∼{\left(\epsilon_{\text{pf}}l\right)}^{2/3} \end{array}$$ (11)

and

$$\begin{array}{c} C_{lll}\left(l\right)∼\epsilon_{\text{pf}}l \end{array}$$ (12)

in agreement with their scaling in Fig 6. The Fourier transform of *C*_*ll*_(*l*) gives the “variance spectrum” $\Sigma_{k}∼\epsilon_{\text{pf}}^{2/3}\;k^{-5/3}$, where *k* is the wave number, which is similar to the famous Kolmogorov spectrum $E_{k}∼\epsilon_{\text{hd}}^{2/3}\;k^{-5/3}$ for the energy cascade in hydrodynamic turbulence [23, 24].

The linear change of $\sigma_{\text{pf}}^{2}$ with time (Fig 7) suggests that the process of protein folding can be considered as Brownian diffusion in the space of folding fluxes **j**(**g**) against the drift flow 〈**j**[**g**(*t*)]〉_**g**_. The diffusion coefficient is determined as $(d\sigma_{\text{pf}}^{2}/dt)/6$ (e.g., [47]), or *ϵ*_pf_/6. Accordingly, the above discussed condition of the constant rate of change of the variance of folding fluxes, which underlies the observed flux scaling, can be restated in more general terms, i.e., as a requirement that the folding fluxes should represent Brownian diffusion with the diffusion coefficient equal to *ϵ*_pf_/6.

**Fig 8:**
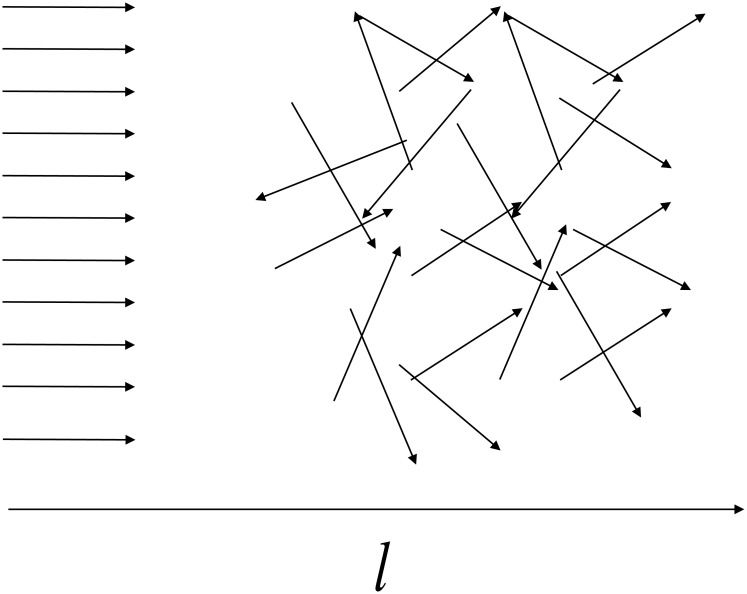
Schematic representation of the transition from a directed flow to a stochastic flow.

The third-order structure function *C*_*lll*_(*l*) in Fig 6**b** is negative. In hydrodynamic turbulence, this corresponds to a direct (Richardson [5]) cascade of eddies, in which large-scale eddies generated by outer forces disintegrate into smaller eddies until the latter dissipate due to viscosity. In more general terms, the negative value of the *C*_*lll*_(*l*) can be associated with the transition from a well-organized (large scale) motion to a stochastic (small scale) motion, as schematically illustrated in Fig 8. As can be seen from this figure, irrespective of whether the initial point is taken in the region of well-directed flow and the terminal point is chosen in the stochastic flow region, or vise versa, the “longitudinal” increment of the flow *δj*_||_(*l*) given by Eq (8) will be negative, and, thus the function *C*_*lll*_(*l*) will also be negative [Eq (7)].

**Fig 9:**
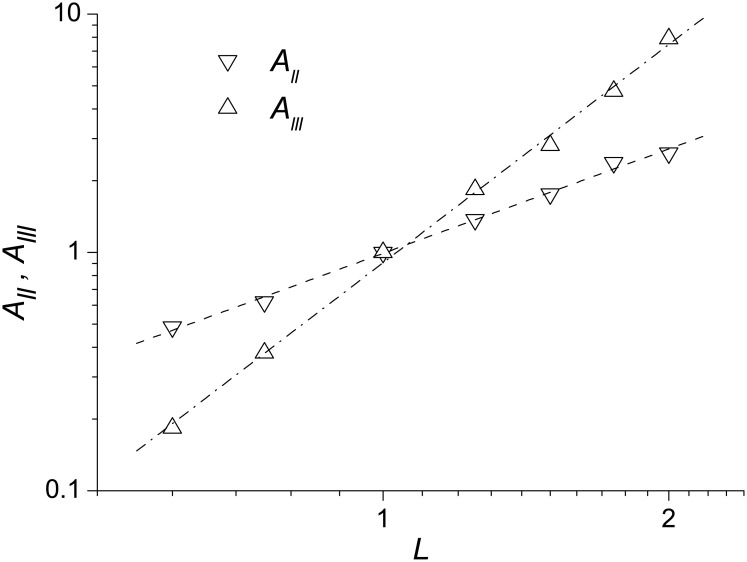
Prefactors *A*_*ll*_ and *A*_*lll*_ determining the structure function scaling with the coarse-graining length *L*. Triangles-down and triangles-up are for the structures of the second and third orders, respectively. The dashed and dash-dot lines represent the best-fits of *A*_*ll*_ and *A*_*lll*_ to the equations *A_ll_* ∼ *L*^*D_ll_*^ and *A_lll_* ∼ *L*^*D_lll_*^ (*D*_*ll*_ ≈ 1.45 and *D*_*lll*_ ≈ 3.0).

Both the function *G*_*k*_(*L*) (Fig 5**a**–5**c**) and the structure functions *C*_*ll*_(*l*) and *C*_*lll*_(*l*) (Fig 6**a** and 6**b**) reveal that the folding flows are self-similar. At the same time, their self-similarities are different in that the *G*_*k*_(*L*) displays a “transversal” self-similarity of the flow distributions, and the structure functions show a “longitudinal” self-similarity. It is thus of interest to see how, and if, the “transversal” and “longitudinal” self-similarities are consistent with each other. Since the flow through a region of size *L* scales with *L* as *J*(*L*) ∼ *L*^*D*^ (Fig 5), and the total volume *V* remains the same at different *L*, $\epsilon_{\text{pf}}\left(L\right)=d\sigma_{\text{pf}}^{2}\left(L\right)/dt∼J{\left(L\right)}^{2}/T∼L^{2D}/T^{3}$, where *T* stands for time. Then, according to Eqs (11) and (12), the second-order structure function should scale with *L* as *C*_*ll*_(*l*, *L*) = *A*_*ll*_(*L*)*C*_*ll*_(*l*, *L*_0_), where *A*_*ll*_(*L*) ∼ *L*^2*D*^, and the third-order structure function as *C*_*lll*_(*l*, *L*) = *A*_*lll*_(*L*)*C*_*lll*_(*l*, *L*_0_), where *A*_*lll*_(*L*) ∼ *L*^3*D*^ (*L*_0_ = 1Å). The calculated relations (Fig 9) show that the exponents in these equations are *D* ≈ 0.73 and *D* ≈ 1 for the second- and third-order structure functions, respectively, which are within the range of variation of the fractal index in Fig 5**a**–5**c** (*D* = 0.7–1.4). Also, these values of *D* correspond better to the region adjacent to the native state, where folding flow is more turbulent.

## Conclusions

Turbulent behavior of protein folding flows was first observed when folding of a SH3 domain was studied [19]. Most surprising was that the folding fluxes in the space of collective variables scaled with the space increment similar to the fluid velocities in the Kolmogorov (K41) theory of isotropic and homogeneous turbulence [23, 24]. In the present paper, to see whether such similarity between folding flows and turbulent fluid flows is specific of the SH3 domain or may be common to proteins, we consider another benchmark system—Trp-cage miniprotein. We have studied its folding in detail recently [32] and found the results in good general agreement with the previous works [25–31]. The Trp-cage miniprotein differs from the SH3 domain essentially, both in the structure and mechanism of folding. In particular, kinetics of Trp-cage folding are single-exponential, while for SH3 domain we had double-exponential kinetics, and turbulence was observed only for slow folding trajectories [19]. Further, the approaches to simulate and characterize the folding process are different. The simulations of Trp-cage folding are performed using an all-atom model (CHARMM program [33]), while for the SH3 domain a coarse-grained representation of the protein was used in the form of C_*α*_-model [19]. Also, in the present case, the collective variables are determined with a PCA method, whereas in the case of SH3 domain they were represented by weakly dependent groups of native contacts [19]. Despite such a considerable difference between the SH3 domain and Trp-cage miniprotein cases, we have found that the structure functions of the second and third orders for the Trp-cage folding follow the Kolmogorov scaling similar to what was observed for the SH3 domain, i.e., *C*_*ll*_(*l*) ∼ *l*^2/3^ and *C*_*lll*_(*l*) ∼ *l*, where *l* is the increment in the space of collective variables. In contrast to classical hydrodynamic turbulence, which considers an incompressible fluid, and thus uses fluid velocities to characterize the flow, we employ flow fluxes because folding fluid is very compressible. In this characterization, the variance of folding fluxes per unit volume $\sigma_{\text{pf}}^{2}\left(g\right)$, where **g** is the point in the three dimensional space of collective variables, plays a role of the kinetic energy of fluctuation per unit mass in hydrodynamic turbulence. The calculation of $\sigma_{\text{pf}}^{2}$ as a function of time has shown that it varies with time essentially linearly, so that the quantity $\epsilon_{\text{pf}}=d\sigma_{\text{pf}}^{2}/dt$ represents the key parameter to characterize the folding flows, similar to the time-rate of change of the kinetic energy per unit mass in hydrodynamic turbulence. In more general terms, the process of protein folding in the space of probability fluxes represents Brownian diffusion (against the drift flow) with the diffusion coefficient equal to *ϵ*_pf_/6. The analysis of the probability flux distribution scaling with the size of coarse-graining of the conformational space has also shown that the distributions are self-similar with a fractal dimension, and the fractal index decreases toward the native state, indicating that the flow becomes more turbulent as the native state is approached.

The obtained results, first, show that the very complex dynamics of protein folding allows a simple characterization in terms of scaling and diffusion of probability fluxes, and, secondly, they suggest that the turbulence phenomena similar to hydrodynamic turbulence are not specific of folding of a particular protein but are common to protein folding.
